# Output stream analysis in a queueing model with working vacation mechanism as a power reduction strategy

**DOI:** 10.1371/journal.pone.0341422

**Published:** 2026-01-27

**Authors:** Martyna Kobielnik, Wojciech M. Kempa

**Affiliations:** Department of Mathematical Methods in Technology and Informatics, Faculty of Applied Mathematics, Silesian University of Technology, Gliwice, Poland; City University of Hong Kong, HONG KONG

## Abstract

The aim of this paper is to analyze the departure process of a queueing model with general independent input stream, exponentially distributed service time, finite buffer and working vacation mechanism. The explicit solution for a Laplace transform of generating function of this characteristic is determined. Utilizing numerical Laplace transform inversion, the time-dependent mean number of jobs served in a time interval is computed. The numerical examples show the behavior of the output stream in dependence on the model parameters, such as interarrival distribution, mean working vacation period duration, the workload in normal and in working vacation mode.

## Introduction

Since the first introduction by Erlang, the queueing models have grown as a very powerful tool to model many real-world phenomena present in informatics, logistics, operation research, and many more fields of science. Their usage enables the analysis of effects that different modeling choices have on, e.g., the cost, energy consumption, and quality of service of the implemented solution.

Recently, the literature on queueing models with different control mechanisms is growing fast. In this paper, the working vacation queue is considered. In traditional vacation queues, when system state approaches 0, the server is turned off for a random amount of time, hence during vacation period, even if a customer appears, no service is offered. Working vacations differ from vacations by still providing the service during the working vacation period, but with smaller speed.

### Queueing models to solve the energy consumption problems

In many cases, the reduction in energy consumption is of great importance. On one hand, in some cases, the usage of devices that are hard to reach to change the energy source causes the need to apply some solution that lowers the energy consumption as much as possible (e.g., sensor networks); on the other hand, there are tools important and widely used nowadays, such as cloud services, that, by their nature, consume enormous amounts of energy. One of possible solutions is to turn the server off when it is idle, i.e., vacation policy. If the traffic of jobs causes too many changes in server’s mode, it is reasonable to apply N-policy, which will minimize the excessive power consumption associated with the server activation.

One can find a great deal of publications that consider vacation queues for the energy usage optimization. In [1], the N-policy vacation model with batch arrivals is considered to provide a power saving algorithm based on the threshold in a node of the Wireless Sensor Network. A vacation priority queue in [2] was analyzed in the context of the energy savings strategy in cloud data centers. The steady-state distribution was obtained with matrix geometric approach, and the quality of service and cost metrics were obtained and solved with the Gauss-Siedel method. In [3] a multi-server Markovian queue with N-policy was applied to study the virtual machine scheduling strategy in cloud data centers.

Putting the server into a sleep mode may cause some issues, e.g. increase of the waiting time, cause the customer impatience, cause the congestion due to faster job accumulation. Furthermore, improving energy efficiency by employing the N-policy to reduce the number of mode changes may, in some circumstances, amplify these drawbacks. To find the compromise between energy savings and quality of service, the working vacation (WV) mode can be applied instead of (or alongside) vacation. In WV mode, instead of putting the server to sleep, the service rate is lowered, hence the queue of customers is served continuously, so the waiting time of a customer that enters during working vacations is lower than for a customer entering during vacation in traditional vacation queue. The examples of energy saving capability of such approach can be found in, e.g. [4], where the energy saving is achieved by frequency and voltage scaling, which results with a slower task processing by the CPU in real-time embedded systems, in [5] where the same approach is used for energy efficiency in cloud computing systems, respectively, or in [6], where one of the considered techniques is slower request processing to save energy in data center storage systems.

The first contribution to WV queues was made in 2002 by Servi and Finn in [7], where an *M*/*M*/1 queue with this mechanism was proposed to model the behavior of the WDM optical access network. Next, this idea was generalized to a *GI*/*M*/1 model in [8], and an *M*/*G*/1 queue in [9].

Since Servi and Finn’s paper, a lot of contributions on the topic of WV have been made in many different settings. In [10] for a Markovian WV queue with setup times, the queue length distribution was obtained using a quasi-birth-and-death process and the geometric matrix approach. Moreover, the stochastic decomposition of the mean waiting time is presented. A *MAP*/*G*/1 model with vacation interruption was analyzed using a supplementary variable and censoring technique to derive the queue length distribution and the Laplace-Stieltjes transform of the waiting time in [11]. The WV queue with general service and vacation interruption under the Bernoulli schedule was considered in [12] to obtain the distribution of the length of the queue and the mean value. The comparison of user utility for different setups of threshold WV policy (exhaustive and non-exhaustive) in a finite buffer Markovian queue was made in [13] with a linear cost-reward function. The Nash equilibrium concept was used in [14] to analyze equilibrium customer strategies in different scenarios in a *M*/*M*/1 queue with balking and retrials. Potential applications to model the performance of media access control function in wireless networks were mentioned. In [15], the generating function was used to obtain the mean performance measures of a Markovian queue with WV and impatient customers. Stochastic decomposition was performed to study the impact of the WV policy on the obtained measures. In [16], the stochastic decomposition for vacation queues (including WV) is surveyed to show how to obtain the additional mean waiting time and mean queue length resulting from the limited server availability. In [17], the supplementary variable technique is presented to analyze a *M*/*G*/1 type queues with service interruption. It is shown how to apply the technique to obtain performance measures, also in the case of a WV queue.

Many papers are devoted to the analysis of cost optimization and energy saving capabilities of WV policy, the following mentioned works do not exhaust the topic. The wake-up scheme for energy saving in wireless sensor networks using a Markovian queue with vacations, working vacations, and N-policy is studied in [18]. Performance measures and energy consumption are derived using generalized Petri nets. An *M*/*M*/*c* queue with vacation mode and threshold WV is applied to the energy usage of the model in [19]. With a modified firefly algorithm, the optimal sleep parameters are found for the problem of virtual machines scheduling in cloud data centers. In [20], the performance measures and the cost-performance ratio are derived and then optimized using particle swarm optimization method for an *M*/*M*/*c* model of a flat semi-dormant multicontroller. In [21], the particle swarm optimization is applied to optimize the cost function derived for a finite buffer *M*/*M*/1 queue with feedback policy. The queue length, balking strategy, and user benefit of a Markovian queue with balking and vacation are studied in [22]. With the quasi-Newton method and the genetic algorithm, the cost function of a Markovian queue with retrial, balking, and imperfect service is optimized in [23]. The average number of jobs in the system and in the orbit are also obtained. In [24], a Markovian model with two-stage service, hybrid vacations, and breakdowns is considered. The cost function derived for the model is optimized with particle swarm optimization and the artificial bee colony algorithm.

### Steady-state vs transient analysis

Most papers on queueing models are devoted to the steady-state analysis. It seems natural since it is much easier and less computationally complex than to find the corresponding time-dependent characteristics for the same model. Moreover, most of the transient solutions are obtained for the simplest *M*/*M*/1 queue. Every single queue control mechanism, such as balking, retrials, breakdowns, vacations, and so on, complicates the study of the model in transient state. On the other hand, in some cases, the steady-state analysis is not sufficient. When one wants to analyze the server in short time after opening, when the behavior of the server or input stream changes over time, when some system parameter needs to be adjusted, or when the convergence to the steady state is very slow, the steady-state characteristics may not reflect the actual behavior of the system. Sometimes, the steady state does not even exist, hence the study of transient characteristics is the only way to study the stochastic behavior of a model.

One can find a survey on approaches to obtain the transient state characteristics in, e.g., [25,26]. In most cases, especially for non-Markovian queues, some approximate methods need to be employed to solve the model. In [27], a diffusion approximation model was used to study a *G*/*G*/1/*N* model with priorities with application to a Software-Defined Network. In [28], the martingale central limit theorem is employed for a queue where arrival and service are both Markov-modulated nonhomogeneous Poisson processes to obtain the diffusion approximation of queue length process. A method to obtain the transient characteristics is described in [29], using a phase-type approximation and presented on a time-inhomogeneous M(t)/M/1 queue with finite buffer. In [30], the transient system size distribution and mean value are found through the Laplace transform for a Markovian queue with vacations and disasters. The queue size distribution for a batch arrival *M*^*X*^/*G*/1/*N* queue with vacations under N-policy is studied in [1] using renewal theory and some algebraic results. The transient waiting time for a *MMAP*/*G*/1/*N* queue with general active queue management scheme is studied in [31].

For a queueing models with WV, transient characteristics are analyzed in, e.g., [32–34].

### Paper contribution

This paper is the continuation of previous work on the *GI*/*M*/1/*N* model with single working vacation, in which various transient characteristics were obtained. The virtual waiting time of a job in [35] provides insight into how long a single job must wait for the service. The distribution of the number of jobs present was analyzed in [36]. This characteristic helps track the usage of the buffer. The time to the first buffer overflow, discussed in [37] shows how long the server works before it starts rejecting jobs due to the buffer saturation.

In this paper, the server performance is analyzed from the perspective of successfully finished jobs. An explicit transient solution for the mean number of jobs finished up to t≥0 is provided and illustrated with numerical examples.

## The model

### Model description

The model under consideration is a *GI*/*M*/1/*N* queue with a single working vacation mode (WV). The input stream is a general independent stream with an interarrival time distribution $F_{A}(t),\;t\geq 0$, and arriving jobs are processed in order of arrival in an exponentially distributed time. There is only one server available, and the buffer space is limited to *N*, so at any time, at most *N* jobs can be present in the system, one in a service unit, and N−1 in the queue. The service speed varies over time. The standard intensity of service is *μ*. Every time the queue is empty after service completion epoch, the server switches to a WV mode, changing the service intensity to a lower one, $\mu_{v}$. After the exponentially distributed with mean 1/α amount of time, the standard service speed is restored.

In the following two sections, the system of integral equations for the output stream is set up using the continuous version of the law of total probability with respect to the first arrival after the server is turned on. It is then rewritten with generating functions and Laplace transforms, in a form that enables the use of a potential-based approach.

The following notation is used: Y(t)=I{server is in WV mode at epoch t}, where I{A} is an indicator function of an event A; X(t) is the state of the system at t≥0; N(t) is the number of jobs finished up to t≥0; $p_{i}(\lambda )=\frac{\lambda^{i}}{i!}e^{-\lambda },\;i=0,1,\ldots$, that is $p_{i}(\lambda )$ is the probability function of a Poisson random variable with parameter λ; $E_{k,\lambda }(t)=1\;-\;\sum_{i=0}^{k-1}p_{k}(\lambda t),\;t\geq 0$, that is $E_{k,\lambda }(t)$ is a CDF of an Erlang distributed random variable with shape parameter *k* and a rate *λ*; $G_{n}(t,m)=P\left(N(t)=m|X(0)=n,Y(0)=0\right),\;t\geq 0,\;n=0,1,\ldots ,N,\;m=0,1,\ldots$, i.e., *G*_*n*_(*t*,*m*) is the probability that *m* jobs are served up to epoch *t*, given that at the opening there were *n* jobs present and the server was in a normal mode; $G_{n}^{*}(t,m)=P\left(N(t)=m|X(0)=n,Y(0)=1\right),\;t\geq 0,\;n=0,1,\ldots ,N,\;m=0,1,\ldots$, where this time the server is in WV mode when it is set up.

### Server starting in normal mode

We start with the server that is in normal mode at *t* = 0. The system of integral equations is built for the probability functions of *m* jobs finished prior to *t*, conditioned by the initial state of the system X(0).

If the server is empty at the opening and no new job enters up to *t*, the only possibility is that no jobs were served in [0,t], hence the second summand in (1). Given that there was a new arrival at epoch 0<*y*<*t*, the probability of *m* jobs served up to *t* is equal to the probability that, starting with one job in normal mode, *m* jobs are served up to t−y, which follows from the fact (which is also used for the remaining equations in this section) that an arrival epoch is a Markov moment of this process. That means that, after every arrival, the stochastic characteristics of this process depend on the system state at the moment of arrival only, i.e., the server can be viewed as it is turned on on the arrival epoch with appropriate number of jobs (i.e., n−k+1, where *n* is the initial value, and *k* is the number of finished jobs prior to the first arrival).

$$G_{0}(t,m)=\int_{0}^{t}G_{1}(t-y,m)dF_{A}(y)+I\left\{m=0\right\}\left(1-F_{A}(t)\right)$$ (1)

For n=1,2,…,N we can write:

$$G_{n}(t,m)=\sum_{i=1}^{3}S_{i}(t,n,m).$$ (2)

The first summand is

$$S_{1}(t,n,m)=\int_{0}^{t}\sum_{k=0}^{n-1}I\left\{m-k\geq 0\right\}p_{k}(\mu y)G_{\min\{n-k+1,N\}}(t-y,m-k)dF_{A}(y),$$ (3)

and follows from the case of a new arrival at y<t, and some (but not all) of the initially present *n* jobs are processed prior to *y*. Obviously, no more than *m* jobs can be served, so that at epoch *t*, the total of *m* jobs left the server. The next summand covers the case of a new arrival at *y*<*t*, and all *n* tasks done before the arrival, so the server switches to WV mode at epoch *u*<*y* when the last task is finished. Therefore, two cases must be covered: the server is still in WV mode at the time of arrival (the WV period is longer than *y*−*u*), or the WV period ends before the first arrival. Therefore, for m≥n,

$$S_{2}(t,n,m)=\int_{0}^{t}\int_{0}^{y}[\left(1-e^{-\alpha (y-u)}\right)G_{1}(t-y,m-n)\;+e^{-\alpha (y-u)}G_{1}^{*}(t-y,m-n)]dE_{n,\mu }(u)dF_{A}(y).$$ (4)

The last summand describes the case of *y*>*t*. To have exactly *m* jobs finished before *t*, we demand X(0)=n≥m, hence

$$S_{3}(t,n,m)=(1-F_{A}(t))(I\left\{n>m\right\}p_{m}(\mu t)+I\left\{n=m\right\}\sum_{k=m}^{\infty }p_{k}(\mu t)).$$ (5)

### Server in WV mode at the opening

If the server begins work in WV mode, the time when the WV period ends needs to be considered. In (6) in the integral, the two summands are for the case where the WV period ends before the new arrival and for the case where the server is still on WV upon arrival, respectively.

$$G_{0}^{*}(t,m)=\int_{0}^{t}[(1-e^{-\alpha y})G_{1}(t-y,m)+e^{-\alpha y}G_{1}^{*}(t-y,m)]dF_{A}(y)+I\left\{m=0\right\}(1-F_{A}(t))$$ (6)

Similarly to *G*_*n*_(*t*,*m*), $G_{n}^{*}(t,m)\;n=1,2,\ldots ,N$ is defined as the sum of several elements to preserve readability:

$$G_{n}^{*}(t,m)=\sum_{i=1}^{6}S_{i}^{*}(t,n,m),$$ (7)

for n=1,2,…,N. In $S_{1}^{*}(t,n,m),\ldots ,S_{4}^{*}(t,n,m),$ we have an arrival at *y*<*t*. The WV period ends before the arrival epoch in $S_{1}^{*}(t,n,m),\ldots ,S_{3}^{*}(t,n,m)$. In the first summand, not all initial present jobs were completed prior to the first arrival, in the second, all *n* tasks left the server before the WV period ended, so no new WV period is initialized, and in the third, the server finished all jobs in normal mode, which activates the WV mode.

$$S_{1}^{*}(t,n,m)=\int_{0}^{t}\int_{0}^{y}\sum_{k=0}^{n-1}p_{k}(\mu^{*}u)\sum_{l=0}^{n-k-1}p_{l}(\mu (y-u))I\left\{m-k-l\geq 0\right\}\;\cdot G_{\min\left\{n-k-l+1,N\right\}}(t-y,m-k-l)\alpha e^{-\alpha u}du\;dF_{A}(y)$$ (8)

$$S_{2}^{*}(t,n,m)=\int_{0}^{t}\int_{0}^{y}\sum_{k=n}^{\infty }p_{k}(\mu^{*}u)I\left\{m-n\geq 0\right\}G_{1}(t-y,m-n)\alpha e^{-\alpha u}\;du\;dF_{A}(y)$$ (9)

$$S_{3}^{*}(t,n,m)=\int_{0}^{t}\int_{0}^{y}\alpha e^{-\alpha u}\sum_{k=0}^{n-1}p_{k}(\mu^{*}u)\int_{0}^{y-u}I\left\{m-n\geq 0\right\}\;\cdot [(1-e^{-\alpha (y-(u+w))})G_{1}(t-y,m-n)+e^{-\alpha (y-(u+w))}G_{1}^{*}(t-y,m-n)]\;\cdot d_{w}E_{n-k,\mu }(w)du\;dF_{A}(y)$$ (10)

In the next summand, at the arrival epoch *y*, the WV mode is still on, so it follows that

$$S_{4}^{*}(t,n,m)=\int_{0}^{t}e^{-\alpha y}[\sum_{k=0}^{n-1}p_{k}(\mu^{*}y)I\left\{m-k\geq 0\right\}G_{\min\{n-k+1,N\}}^{*}(t-y,m-k)\;+\sum_{k=n}^{\infty }p_{k}(\mu^{*}y)I\left\{m-n\geq 0\right\}G_{1}^{*}(t-y,m-n)]dF_{A}(y)$$ (11)

The last two summands reflect the case of no new arrivals before *t*. In $S_{5}^{*}(t,n,m),$ the WV mode ends before *t*, while in $S_{6}^{*}(t,n,m),$ it is still active at the epoch *t*.

$$S_{5}^{*}(t,n,m)=(1-F_{A}(t))\int_{0}^{t}\alpha e^{-\alpha u}[\sum_{k=0}^{n-1}p_{k}(\mu^{*}u)\sum_{l=0}^{n-k-1}p_{l}(\mu (t-u))I\left\{m=k+l\right\}\;+I\left\{m=n\right\}\left(\sum_{k=0}^{n-1}p_{k}(\mu^{*}u)\sum_{l=n-k}^{\infty }p_{l}(\mu (t-u))+\sum_{k=n}^{\infty }p_{k}(\mu^{*}u)\right)]du$$ (12)

$$S_{6}^{*}(t,n,m)=(1-F_{A}(t))e^{-\alpha t}(\sum_{k=0}^{n-1}p_{k}(\mu^{*}t)I\left\{m=k\right\}+\sum_{k=n}^{\infty }p_{k}(\mu^{*}t)I\left\{m=n\right\})$$ (13)

## Solution

In the first part of this section, the model is stated in terms of the Laplace transforms of the generating functions of sequences $G_{n}(t,m),\;G_{n}^{*}(t,m),\;m=0,1,\ldots$. Then, the solution is found in two steps: solving the case of server starting in normal mode, which, as will be seen, depends on the solution of $G_{n}^{*}(t,m)$, and then solving the second part, which concludes in an explicit solution.

To state the system in the desired form, the following steps are performed:

1. Introduce the generating functions for n=0,…,N:
  $${\tilde{G}}_{n}(t,z)=\sum_{m=0}^{\infty }z^{m}G_{n}(t,m),\;{\tilde{S}}_{i}(t,n,z)=\sum_{m=0}^{\infty }z^{m}S_{i}(t,n,m),\;i=1,2,3,\;{\tilde{G}}_{n}^{*}(t,z)=\sum_{m=0}^{\infty }z^{m}G_{n}^{*}(t,m),\;{\tilde{S}}_{i}^{*}(t,n,z)=\sum_{m=0}^{\infty }z^{m}S_{i}^{*}(t,n,m),\;i=1,\ldots ,6$$
  The equations *G*_*n*_(*t*,*m*) ($G_{n}^{*}(t,m)$, respectively) are multiplied by *z*^*m*^ and summed over m=0,1,…, yielding ${\tilde{G}}_{n}(t,z)$ (${\tilde{G}}_{n}^{*}(t,z)$).
2. Introduce the Laplace transforms for n=0,…,N:
  $${\bar{G}}_{n}(s,z)=\int_{0}^{\infty }e^{-ts}{\tilde{G}}_{n}(t,z)dt,\;{\bar{S}}_{i}(s,n,z)=\int_{0}^{\infty }e^{-st}{\tilde{S}}_{i}(t,n,z)dt,i=1,2,3\;{\bar{G}}_{n}^{*}(s,z)=\int_{0}^{\infty }e^{-ts}{\tilde{G}}_{n}^{*}(t,z)dt,\;{\bar{S}}_{i}^{*}(s,n,z)=\int_{0}^{\infty }e^{-st}{\tilde{S}}_{i}^{8}(t,n,z)dt,i=1,\ldots ,6$$
  Taking the Laplace transforms of the left and right sides of the equations yields the form of ${\tilde{G}}_{n}(s,z)$ and ${\tilde{G}}_{n}^{*}(s,z)$.

Later on, the following notation is used for the readability:


$${\bar{F}}_{A}(s)=\int_{0}^{\infty }e^{-st}F_{A}(t)dt,\;{\bar{F}}_{A}^{S}(s)=\int_{0}^{\infty }e^{-st}dF_{A}(t)=s{\bar{F}}_{A}(s)$$

$$a_{i}(s,z)=z^{i}\int_{0}^{\infty }e^{-st}p_{i}(\mu t)dF_{A}(t)$$ (14)

$$b_{i}(s,z)=z^{i}\int_{0}^{\infty }e^{-st}E_{i,\mu }(t)dF_{A}(t)$$ (15)

$$c_{i}(s,z)={\left(\frac{z\mu }{\mu -\alpha }\right)}^{i}\int_{0}^{\infty }e^{-t(s+\alpha )}E_{i}((\mu -\alpha )t)dF_{A}(t)$$ (16)

$$a_{i}^{*}(s,z)=z^{i}\int_{0}^{\infty }e^{-t(s+\alpha )}p_{i}(\mu^{*}t)dF_{A}(t)$$ (17)

$$b_{i}^{*}(s,z)=z^{i}\sum_{k=i}^{\infty }\int_{0}^{\infty }e^{-t(s+\alpha )}p_{k}(\mu^{*}t)dF_{A}(t)$$ (18)

$$c_{i}^{*}(s,z)=z^{i}\sum_{k=0}^{i-1}\int_{0}^{\infty }e^{-st}\int_{0}^{t}\alpha e^{-\alpha u}p_{k}(\mu^{*}u)E_{i-k,\mu }(t-u)du\;dF_{A}(t)$$ (19)

$$d_{i}^{*}(s,z)=z^{i}\sum_{k=0}^{i-1}{\left(\frac{\mu }{\mu -\alpha }\right)}^{i-k}\int_{0}^{\infty }\alpha e^{-t(s+\alpha )}\int_{0}^{t}p_{k}(\mu^{*}u)E_{i-k,\mu -\alpha }(t-u)du\;dF_{A}(t)$$ (20)

$$A_{ij}(s,z)=z^{i+j}\int_{0}^{\infty }e^{-st}\int_{0}^{t}\alpha e^{-\alpha u}p_{i}(\mu^{*}u)p_{j}(\mu (t-u))du\;dF_{A}(t)$$ (21)

$$B_{i}(s,z)=z^{i}\sum_{k=i}^{\infty }\int_{0}^{\infty }e^{-st}\int_{0}^{t}\alpha e^{-\alpha u}p_{k}(\mu^{*}u)du\;dF_{A}(t)$$ (22)

With the introduced notation, we can express the system (1), (2), (6), (7) in the form

$${\bar{G}}_{0}(s,z)=s{\bar{F}}_{A}(s){\bar{G}}_{1}(s,z)+\frac{1}{s}-{\bar{F}}_{A}(s),$$ (23)

$${\bar{G}}_{n}(s,z)=\sum_{i=1}^{3}{\bar{S}}_{i}(s,n,z),$$ (24)

$${\bar{G}}_{0}^{*}(s,z)={\bar{G}}_{1}(s,z)s\left({\bar{F}}_{A}(s)-{\bar{F}}_{A}(s+\alpha )\right)+{\bar{G}}_{1}^{*}(s,z)s{\bar{F}}_{A}(s+\alpha )+\frac{1}{s}-{\bar{F}}_{A}(s),$$ (25)

$${\bar{G}}_{n}^{*}(s,z)=\sum_{i=1}^{6}{\bar{S}}_{i}^{*}(s,n,z),$$ (26)

for n=1,2,…,N, where

$${\bar{S}}_{1}(s,n,z)=\sum_{k=0}^{n-1}a_{k}(s,z){\bar{G}}_{\min\{n-k+1,N\}}(s,z),$$ (27)

$${\bar{S}}_{2}(s,n,z)=b_{n}(s,z){\bar{G}}_{1}(s,z)+c_{n}(s,z)({\bar{G}}_{1}^{*}(s,z)-{\bar{G}}_{1}(s,z)),$$ (28)

$${\bar{S}}_{3}(s,n,z)=\sum_{k=0}^{n-1}z^{k}\int_{0}^{\infty }e^{-st}(1-F_{A}(t))p_{k}(\mu t)dt+z^{n}\sum_{k=n}^{\infty }\int_{0}^{\infty }e^{-st}(1-F_{A}(t))p_{k}(\mu t)dt,$$ (29)

$${\bar{S}}_{1}^{*}(s,n,z)=\sum_{k=0}^{n-1}\sum_{l=0}^{n-k-1}A_{kl}(s,z){\bar{G}}_{\min\{n-(k+l)+1,N\}}(s,z),$$ (30)

$${\bar{S}}_{2}^{*}(s,n,z)=B_{n}(s,z){\bar{G}}_{1}(s,z),$$ (31)

$${\bar{S}}_{3}^{*}(s,n,z)=c_{n}^{*}(s,z){\bar{G}}_{1}(s,z)+d_{n}^{*}(s,z)({\bar{G}}_{1}^{*}(s,z)-{\bar{G}}_{1}(s,z)),$$ (32)

$${\bar{S}}_{4}^{*}(s,n,z)=\sum_{k=0}^{n-1}a_{k}^{*}(s,z){\bar{G}}_{\min\{n-k+1,N\}}^{*}(s,z)+b_{n}^{*}(s,z){\bar{G}}_{1}^{*}(s,z),$$ (33)

$${\bar{S}}_{5}^{*}(s,n,z)=\sum_{k=0}^{n-1}\sum_{l=0}^{n-k-1}z^{k+l}\int_{0}^{\infty }e^{-st}(1-F_{A}(t))\int_{0}^{t}p_{k}(\mu^{*}u)p_{l}(\mu (t-u))\alpha e^{-\alpha u}du\;dt\;+z^{n}\sum_{k=0}^{n-1}\sum_{l=0}^{n-k-1}\int_{0}^{\infty }e^{-st}(1-F_{A}(t))\int_{0}^{t}p_{k}(\mu^{*}u)p_{l}(\mu (t-u))\alpha e^{-\alpha u}du\;dt\;+z^{n}\sum_{k=n}^{\infty }\int_{0}^{\infty }e^{-st}(1-F_{A}(t))\int_{0}^{t}p_{k}(\mu^{*}u)\alpha e^{-\alpha u}du,$$ (34)

$${\bar{S}}_{6}^{*}(s,n,z)=\sum_{k=0}^{n-1}z^{k}\int_{0}^{\infty }e^{-t(s+\alpha )}(1-F_{A}(t))p_{k}(\mu^{*}t)dt+z^{n}\sum_{k=n}^{\infty }\int_{0}^{\infty }e^{-t(s+\alpha )}(1-F_{A}(t))p_{k}(\mu^{*}t)dt.$$ (35)

Now we are ready to solve for ${\bar{G}}_{n}(s,z),\;{\bar{G}}_{n}^{*}(s,z)$. The following theorem is used for this purpose (see [38]):

**Theorem 1.** *Each solution of the infinite-size system of linear equations of the form*

$$\sum_{k=-1}^{n-2}a_{k+1}x_{n-k}-x_{n}=\theta_{n},$$ (36)

*where* n≥2, *can be written as*

$$x_{n}=MR_{n-1}+\sum_{k=2}^{n}R_{n-k}\phi_{k},\;n\geq 2,$$ (37)

*where* M∈ℝ *and the sequence (R_k_) can be defined recursively as follows:*

$$R_{0}=0,\;R_{1}=(a_{0}{)}^{-1},\;R_{k}=R_{1}\left(R_{k-1}-\sum_{j=0}^{k-1}a_{j+1}R_{k-1-i}\right),$$ (38)

*where* k≥2.

### Solution for normal mode at opening

The system (24) can be rewritten for n=1,2,…,N−1 as follows:

$$\sum_{k=-1}^{n-2}a_{k+1}(s,z){\bar{G}}_{n-k}(s,z)-{\bar{G}}_{n}(s,z)=\phi_{n}(s,z),$$ (39)

where

$$\phi_{n}(s,z)=(c_{n}(s,z)-b_{n}(s,z)){\bar{G}}_{1}(s,z)-c_{n}(s,z){\bar{G}}_{1}^{*}(s,z)-S_{3}(s,n,z).$$ (40)

Utilizing Theorem 1 results in a solution to the system (39) in the form:

$${\bar{G}}_{n}(s,z)=M(s,z)R_{n-1}(s,z)+\sum_{k=2}^{n}R_{n-k}(s,z)\phi_{k}(s,z),$$ (41)

for n≥2, where M(s,z) is some function of *s* and *z* (which is a consequence of the fact that *G*_*n*_(*s*,*z*) is a function of *s* and *z*), and *R*_*n*_(*s*,*z*) is a sequence defined recursively as follows:

$$R_{0}(s,z)=0,\;R_{1}(s,z)=\frac{1}{a_{0}(s,z)},\;R_{k+1}(s,z)=R_{1}(s,z)\left(R_{k}(s,z)-\sum_{i=0}^{k}a_{i+1}(s,z)R_{k-i}(s,z)\right).$$ (42)

Only one solution from (41)is needed. The right solution can be found using the Eq. (24) for *n* = *N* as a boundary condition. Firstly, we need to find M(s,z). Combining (41) for *n* = 2 and (24) for *n* = 1 gives

$$M(s,z)={\bar{G}}_{1}(s,z)+\phi_{1}(s,z).$$ (43)

Introducing the former expression to (41) yields

$${\bar{G}}_{n}(s,z)={\bar{G}}_{1}(s,z)R_{n-1}(s,z)+\sum_{k=1}^{n}R_{n-k}(s,z)\phi_{k}(s,z)$$ (44)

Finally, to satisfy the boundary condition, we combine (24) and (44), both for *n* = *N*, and obtain

$${\bar{G}}_{1}(s,z)=\frac{K_{1}(s,z)}{L(s,z)}{\bar{G}}_{1}^{*}(s,z)+\frac{K_{2}(s,z)}{L(s,z)},$$ (45)

where

$$K_{1}(s,z)=\sum_{k=1}^{N-1}a_{k}(s,z)\sum_{l=1}^{N-k+1}R_{N-k-l+1}(s,z)c_{l}(s,z)+a_{0}(s,z)\sum_{l=1}^{N}R_{N-l}(s,z)c_{l}(s,z)\;-c_{N}(s,z)-\sum_{k=1}^{N}R_{N-k}(s,z)c_{k}(s,z)$$ (46)

$$K_{2}(s,z)=\sum_{k=1}^{N-1}a_{k}(s,z)\sum_{l=1}^{N-k+1}R_{N-k-l+1}(s,z)S_{3}(s,l,z)+a_{0}(s,z)\sum_{l=1}^{N}R_{N-l}(s,z)S_{3}(s,l,z)\;-\sum_{k=1}^{N}R_{N-k}(s,z)S_{3}(s,k,z)-S_{3}(s,N,z)$$ (47)

$$L(s,z)=\sum_{k=1}^{N-1}a_{k}(s,z)(R_{N-k}(s,z)+\sum_{l=1}^{N-k+1}R_{N-k-l+1}(s,z)(c_{l}(s,z)-b_{l}(s,z)))\;+a_{0}(s,z)(R_{N-1}(s,z)+\sum_{l=1}^{N}R_{N-l}(s,z)(c_{l}(s,z)-b_{l}(s,z)))\;+b_{N}(s,z)-c_{N}(s,z)+R_{N-1}(s,z)-\sum_{k=1}^{N}R_{N-k}(s,z)(c_{k}(s,z)-b_{k}(s,z))$$ (48)

When solution (45) is applied in (44) for n=2,…,N, as the result, we have

$${\bar{G}}_{n}(s,z)={\bar{G}}_{1}^{*}(s,z)(\frac{K_{1}(s,z)}{L(s,z)}R_{n-1}(s,z)+\sum_{k=1}^{n}R_{n-k}(s,z)(\frac{K_{1}(s,z)}{L(s,z)}(c_{k}(s,z)\;-b_{k}(s,z))-c_{k}(s,z)))+\frac{K_{2}(s,z)}{L(s,z)}R_{n-1}(s,z)+\sum_{k=1}^{n}R_{n-k}(s,z)\;\cdot (\frac{K_{2}(s,z)}{L(s,z)}(c_{k}(s,z)-b_{k}(s,z))-S_{3}(s,k,z))$$ (49)

Note, that the only unknown term in (45) and (49) is ${\bar{G}}_{1}^{*}(s,z)$, which is to be found in the next section.

### Solution for server in WV mode at opening

In this section, the solution for the server starting in WV mode is found. The first step is to introduce the solution (45) and (49) to system (26). As a consequence, for n=1,…,N−1, we have the following form of this system:

$${\bar{G}}_{n}^{*}(s,z)=\sum_{k=0}^{n-1}a_{k}^{*}(s,k){\bar{G}}_{n-k+1}^{*}(s,z)+{\bar{G}}_{1}^{*}(s,z)C_{n}(s,z)+D_{n}(s,z),$$ (50)

and

$${\bar{G}}_{N}^{*}(s,z)=\sum_{k=1}^{N-1}a_{k}^{*}(s,k){\bar{G}}_{N-k+1}^{*}(s,z)+D_{N}(s,z)+a_{0}^{*}(s,z){\bar{G}}_{N}^{*}(s,z)+{\bar{G}}_{1}^{*}(s,z)C_{N}(s,z),$$ (51)

where

$$C_{n}(s,z)=b_{n}^{*}(s,z)+d_{n}^{*}(s,z)+\frac{K_{1}(s,z)}{L(s,z)}\left(c_{n}^{*}(s,z)-d_{n}^{*}(s,z)+B_{n}(s,z)\right)\;+\sum_{k=0}^{n-1}\sum_{l=0}^{n-k-1}A_{kl}(s,z)[\frac{K_{1}(s,z)}{L(s,z)}R_{n-k-l}(s,z)+\sum_{m=1}^{n-k-l+1}R_{n-k-l-m+1}(s,z)\;\cdot \left(\frac{K_{1}(s,z)}{L(s,z)}(c_{m}(s,z)-b_{m}(s,z))-c_{m}(s,z)\right)]$$ (52)

$$D_{n}(s,z)=S_{5}^{*}(s,n,z)+S_{6}^{*}(s,n,z)+\frac{K_{2}(s,z)}{L(s,z)}\left(c_{n}^{*}(s,z)-d_{n}^{*}(s,z)+B_{n}(s,z)\right)\;+\sum_{k=0}^{n-1}\sum_{l=0}^{n-k-1}A_{kl}(s,z)[\frac{K_{2}(s,z)}{L(s,z)}R_{n-k-l}(s,z)+\sum_{m=1}^{n-k-l+1}R_{n-k-l-m+1}(s,z)\;\cdot \left(\frac{K_{2}(s,z)}{L(s,z)}(c_{m}(s,z)-b_{m}(s,z))-S_{3}(s,m,z)\right)]$$ (53)

and for *n* = *N*

$$C_{N}(s,z)=b_{N}^{*}(s,z)+d_{N}^{*}(s,z)+\frac{K_{1}(s,z)}{L(s,z)}\left(c_{N}^{*}(s,z)-d_{N}^{*}(s,z)+B_{N}(s,z)\right)\;+\sum_{k=1}^{N-1}\sum_{l=0}^{N-k-1}A_{kl}(s,z)[\frac{K_{1}(s,z)}{L(s,z)}R_{N-k-l}(s,z)\;+\sum_{m=1}^{N-k-l+1}R_{N-k-l-m+1}(s,z)\left(\frac{K_{1}(s,z)}{L(s,z)}(c_{m}(s,z)-b_{m}(s,z))-c_{m}(s,z)\right)]\;+\sum_{l=1}^{N-1}A_{0l}(s,z)[\frac{K_{1}(s,z)}{L(s,z)}R_{N-l}(s,z)\;+\sum_{m=1}^{N-l+1}R_{N-l-m+1}(s,z)\left(\frac{K_{1}(s,z)}{L(s,z)}(c_{m}(s,z)-b_{m}(s,z))-c_{m}(s,z)\right)]\;+A_{00}(s,z)[\frac{K_{1}(s,z)}{L(s,z)}R_{N-1}(s,z)\;+\sum_{m=1}^{N}R_{N-m}(s,z)\left(\frac{K_{1}(s,z)}{L(s,z)}(c_{m}(s,z)-b_{m}(s,z))-c_{m}(s,z)\right)]$$ (54)

$$D_{N}(s,z)=S_{5}^{*}(s,N,z)+S_{6}^{*}(s,N,z)+\frac{K_{2}(s,z)}{L(s,z)}\left(c_{N}^{*}(s,z)-d_{N}^{*}(s,z)+B_{N}(s,z)\right)\;+\sum_{k=1}^{N-1}\sum_{l=0}^{N-k-1}A_{kl}(s,z)[\frac{K_{2}(s,z)}{L(s,z)}R_{N-k-l}(s,z)\;+\sum_{m=1}^{N-k-l+1}R_{N-k-l-m+1}(s,z)\left(\frac{K_{2}(s,z)}{L(s,z)}(c_{m}(s,z)-b_{m}(s,z))-S_{3}(s,m,z)\right)]\;+\sum_{l=1}^{N-1}A_{0l}(s,z)[\frac{K_{2}(s,z)}{L(s,z)}R_{N-l}(s,z)\;+\sum_{m=1}^{N-l+1}R_{N-l-m+1}(s,z)\left(\frac{K_{2}(s,z)}{L(s,z)}(c_{m}(s,z)-b_{m}(s,z))-S_{3}(s,m,z)\right)]\;+A_{00}(s,z)[\frac{K_{2}(s,z)}{L(s,z)}R_{N-1}(s,z)\;+\sum_{m=1}^{N}R_{N-m}(s,z)\left(\frac{K_{2}(s,z)}{L(s,z)}(c_{m}(s,z)-b_{m}(s,z))-S_{3}(s,m,z)\right)]$$ (55)

For n=1,…,N−1, we rewrite the system (50) in the form

$$\sum_{k=-1}^{n-2}a_{k+1}^{*}(s,z){\bar{G}}_{n-k}^{*}(s,z)-{\bar{G}}_{n}^{*}(s,z)=\phi_{n}^{*}(s,z),$$ (56)

where

$$\phi_{n}^{*}(s,z)=-C_{n}(s,z){\bar{G}}_{1}^{*}(s,z)-D_{n}(s,z).$$ (57)

The form of (56) allows the use of Theorem 1, hence the solution for n≥2 is

$${\bar{G}}_{n}^{*}(s,z)=M^{*}(s,z)R_{n-1}^{*}(s,z)+\sum_{k=2}^{n}R_{n-k}^{*}(s,z)\phi_{k}^{*}(s,z),$$ (58)

where M^*^(*s*,*z*) is some function, and $R_{k}^{*}(s,z)$ is a sequence defined recursively

$$R_{0}^{*}(s,z)=0,\;R_{1}^{*}(s,z)=\frac{1}{a_{0}^{*}(s,z)},\;R_{k+1}^{*}(s,z)=R_{1}^{*}(s,z)(R_{k}^{*}(s,z)-\sum_{i=0}^{k}a_{i+1}^{*}(s,z)R_{k-i}^{*}(s,z))$$ (59)

Combining (58) for *n* = 2 with (50) for *n* = 1 yields:

$$M^{*}(s,z)={\bar{G}}_{1}^{*}(s,z)+\phi_{1}^{*}(s,z)$$ (60)

M^*^(*s*,*z*) is then introduced to (58). The next step is to combine (58) for *n* = *N* and (51), that is, to use the boundary condition, which produces:

$${\bar{G}}_{1}^{*}(s,z)=\frac{K^{*}(s,z)}{L^{*}(s,z)},$$ (61)

where

$$K^{*}(s,z)=(1-a_{0}^{*}(s,z))\sum_{m=1}^{N}R_{N-m}^{*}(s,z)D_{m}(s,z)+D_{N}(s,z)\;-\sum_{k=1}^{N-1}a_{k}^{*}(s,z)\sum_{m=1}^{N-k+1}R_{N-k-m+1}^{*}(s,z)D_{m}(s,z)$$ (62)

$$L^{*}(s,z)=(1-a_{0}^{*}(s,z))(R_{N-1}^{*}(s,z)-\sum_{m=1}^{N}R_{N-m}^{*}(s,z)C_{m}(s,z))-C_{N}(s,z)\;-\sum_{k=1}^{N-1}a_{k}^{*}(s,z)(R_{N-k}^{*}(s,z)-\sum_{m=1}^{N-k+1}R_{N-k-m+1}^{*}(s,z)C_{m}(s,z))$$ (63)

### The final solution

To obtain the final, explicit solution for the Laplace transform of a generating function of a number of jobs served, conditioned by the initial mode and the number of jobs in the system, we induce the expression (61) to (45) and (49), which yields:

$${\bar{G}}_{0}(s,z)=\frac{1}{s}-{\bar{F}}_{A}(s)+s{\bar{F}}_{A}(s)\left(\frac{K^{*}(s,z)}{L^{*}(s,z)}\cdot \frac{K_{1}(s,z)}{L(s,z)}+\frac{K_{2}(s,z)}{L(s,z)}\right)$$ (64)

$${\bar{G}}_{1}(s,z)=\frac{K^{*}(s,z)}{L^{*}(s,z)}\cdot \frac{K_{1}(s,z)}{L(s,z)}+\frac{K_{2}(s,z)}{L(s,z)}$$ (65)

$${\bar{G}}_{n}(s,z)=\;\left(\frac{K^{*}(s,z)}{L^{*}(s,z)}\cdot \frac{K_{1}(s,z)}{L(s,z)}+\frac{K_{2}(s,z)}{L(s,z)}\right)\;\cdot (R_{n-1}(s,z)+\sum_{k=1}^{n}R_{n-k}(s,z)(c_{k}(s,z)-b_{k}(s,z)))\;-\sum_{k=1}^{n}R_{n-k}(s,z)\left(\frac{K^{*}(s,z)}{L^{*}(s,z)}c_{k}(s,z)+S_{3}(s,k,z)\right).$$ (66)

$${\bar{G}}_{0}^{*}(s,z)=\;\left(\frac{K^{*}(s,z)}{L^{*}(s,z)}\frac{K_{1}(s,z)}{L(s,z)}+\frac{K_{2}(s,z)}{L(s,z)}\right)\left(s{\bar{F}}_{A}(s)-s{\bar{F}}_{A}(s+\alpha )\right)\;+\frac{K^{*}(s,z)}{L^{*}(s,z)}s{\bar{F}}_{A}(s+\alpha )+\frac{1}{s}-{\bar{F}}_{A}(s)$$ (67)

$${\bar{G}}_{1}^{*}(s,z)=\frac{K^{*}(s,z)}{L^{*}(s,z)}$$ (68)

$${\bar{G}}_{n}^{*}(s,z)=\frac{K^{*}(s,z)}{L^{*}(s,z)}(R_{n-1}^{*}(s,z)-\sum_{k=1}^{n}R_{n-k}^{*}(s,z)C_{k}(s,z))\;\;-\sum_{k=1}^{n}R_{n-k}^{*}(s,z)D_{k}(s,z)$$ (69)

It is clear, that *G*_*n*_(*s*,*z*) and $G_{n}^{*}(s,z)$ now depend only on the known model parameters. This solution is written for the Laplace transforms of the generating functions of the conditional number of finished jobs distribution. To obtain the distributions, some inversion methods need to be applied to characterize the model properly. Methods used for the purpose of this paper are described in next section.

## Numerical study

In this section, the numerical study is carried out. The characteristic of interest is the mean number of jobs finished up to t, given *n* jobs were present at *t* = 0 *Z*_*n*_(*t*) for system starting in normal, and $Z_{n}^{*}(t)$ in WV mode.

To process with the numerical study, the Laplace transform must be inversed. For the purpose of this paper, the numerical Gaver-Whynn rho inversion algorithm was used, which is easy to use, yet quite precise (see [39]). The resulting generating function can be also inversed with a suitable method (e.g., [40]) to obtain the distribution. We only analyze the mean value, so the well known formula was utilized for computations:


$$E(X)=P_{X}^{\prime }(1),$$

where *P*_*X*_(*z*) is a generating function of a random variable *X*. Further, four examples are presented to analyze the impact of the model parameters on the examined characteristic.

The probability distributions of the time between successive arrivals used for the purpose of numerical examples are presented in the Table 1.

**Table 1:** Random variables used in numerical examples.

	Distribution	Parameters	Coefficient of variation
*X*_1_	Exponential	rate: 15	1
*X*_2_	Uniform	[0;2/15]	0.57
*X*_3_	Gamma	scale: 1/5, shape: 1/3	1.73
*X*_4_	Pareto	scale: 0.05, shape: 4	0.35
*X*_5_	Hyperexponential	$p_{1}=p_{2}=1/2,\;{rate}_{1}=10,\;{rate}_{2}=30$	1.22

The parameters of those distributions were chosen so the input stream has the same intensity of λ=15 in every case. This way, the impact of the value of the coefficient of variation can be observed (see Example 4). Note, that since the arrival stream is determined in all examples, the mean number of jobs that enter or try to but are rejected up to *t* is equal to λt (75 in most examples). The server workload is given by ρ=λ/μ for the normal, and by $\rho^{*}=\lambda /\mu^{*}$ in WV mode.

**Fig 1:**
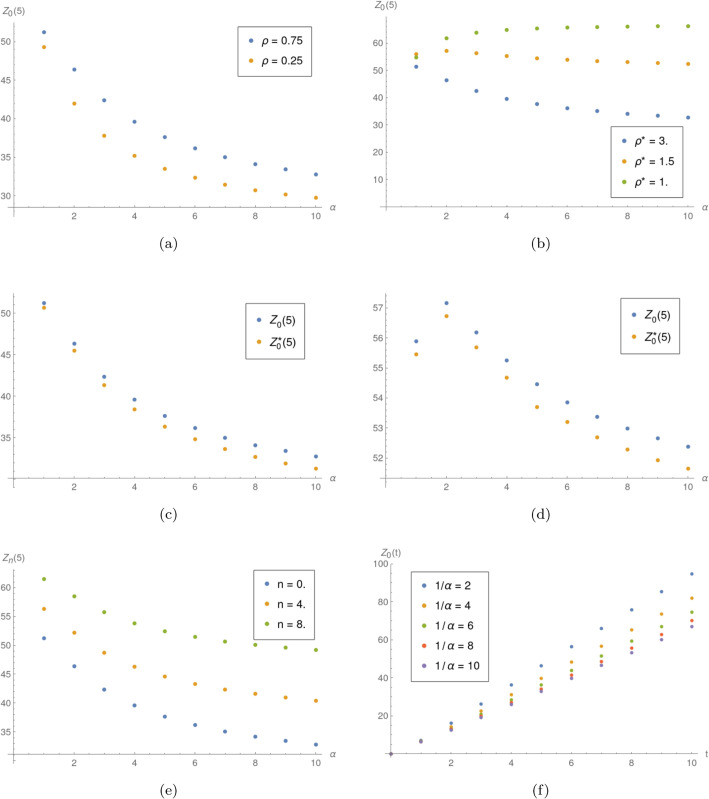
Influence of *α.* a) *Z*_0_(5) as function of 1/α for different values of *ρ*. b) *Z*_0_(5) as function of 1/α for different values of $\rho^{*}$. c) *Z*_0_(5) and $Z_{0}^{*}(5)$ as functions of 1/α for $\rho^{*}=3$. d) *Z*_0_(5) and $Z_{0}^{*}(5)$ as functions of 1/α for $\rho^{*}=1.5$. e) *Z*_*n*_(5) as function of 1/α for different values of *n*. f) *Z*_0_(*t*) for different mean WV period durations 1/α.

**Example 1.** In this example, the impact of *α* is studied. If not stated otherwise, the service rates are μ=20 and $\mu^{*}=5$, which yields ρ=0.75 and $\rho^{*}=3$ for the workload in normal and WV mode, respectively. The interarrival times are uniformly distributed (random variable *X*_2_). The Fig 1 shows the results of experiments. All tests showed that as the mean WV period duration 1/α grows, the mean number of jobs finished in given time period tends to decrease, with some exceptions.

For different values of *ρ* (Fig 1a) it is clear that the decrease is slower for larger values of *ρ*. It is the consequence of the fact that, with the bigger workload, the server will less often switch to the slower service speed. Different observations can be made in regard to $\rho^{*}$ (Fig 1b). When the server is in the WV, the higher the load, the more jobs will be rejected on arrival, hence, less jobs will finally be counted as finished.

In the Fig 1c and 1d, we can notice slightly different behavior of the server when compared the server starting in normal and in WV mode.

The Fig 1e shows how the impact of *α* changes when a different initial number of jobs is used. We can note, that with more initially present jobs, the impact of *α* is slightly less evident because the more jobs present at *t* = 0, the more time (on average) will pass before the first mode switch.

The Fig 1f shows how differently the mean number of finished tasks grows, when the mean time spent in WV mode changes.

**Fig 2:**
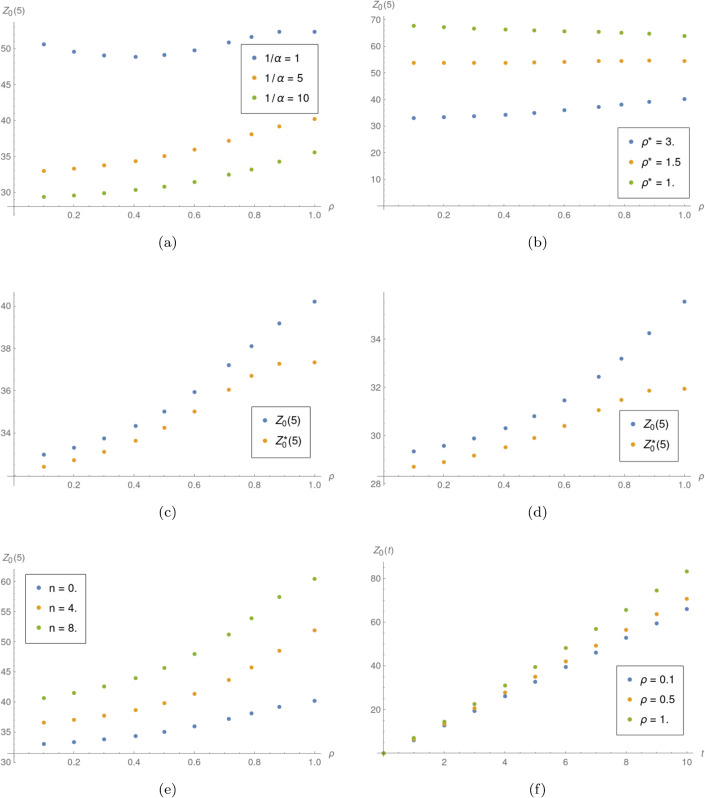
Influence of *ρ.* a) *Z*_0_(5) as function of *ρ* for different values of 1/α. b) *Z*_0_(5) as function of *ρ* for different values of $\rho^{*}$. c) *Z*_0_(5) and $Z_{0}^{*}(5)$ as functions of *ρ* for 1/α=5. d) *Z*_0_(5) and $Z_{0}^{*}(5)$ as functions of *ρ* for 1/α=10. e) *Z*_*n*_(5) as function of *ρ* for different values of *n*. f) *Z*_0_(*t*) for different values of *ρ*.

**Example 2.** Results of the impact of *ρ* investigation are presented in the Fig 2. When no other information is given, the parameters used are 1/α=5 and $\mu^{*}=5$. For the arrival process, random variable *X*_2_ is used. One can note that increasing the server utilization in normal mode leads to higher mean number of jobs served in most cases. This behavior seems natural, since the server are less likely to switch to the lower service speed when *ρ* is bigger. This is also the reason why *Z*_0_(*t*) grows faster over time for bigger *ρ* (see Fig 2f).

**Fig 3:**
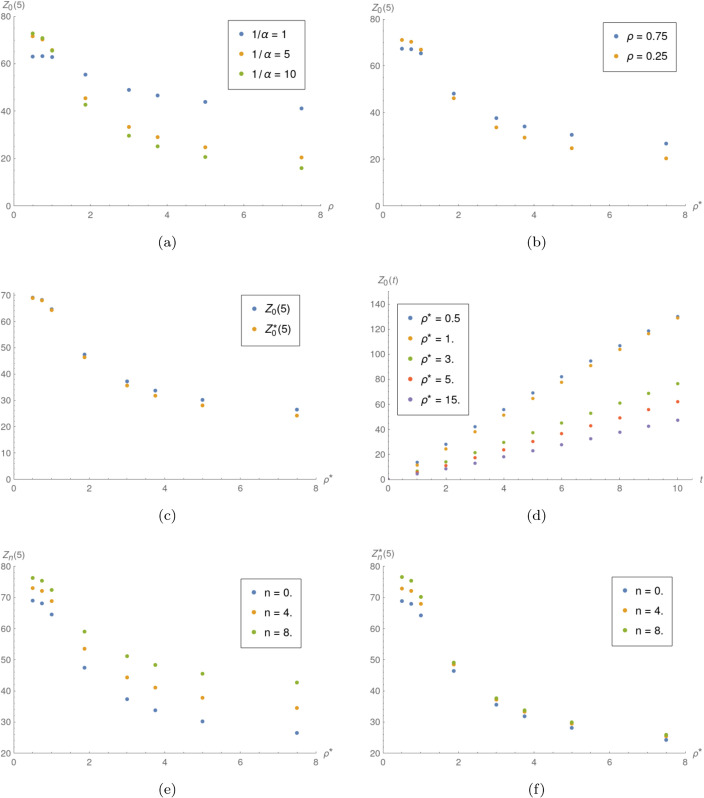
Influence of $\rho^{*}$. a) *Z*_0_(5) as function of $\rho^{*}$ for different values of 1/α. b) *Z*_0_(5) as function of $\rho^{*}$ for different values of *ρ*. c) *Z*_0_(5) and $Z_{0}^{*}(5)$ as functions of $\rho^{*}$. d) *Z*_0_(*t*) for different values of $\rho^{*}$. e) *Z*_*n*_(5) as function of $\rho^{*}$ for different values of *n*. f) $Z_{n}^{*}(5)$ as function of rho^*^ for different values of *n*.

**Example 3.** In this example, the impact of $\rho^{*}$ is investigated. The interarrival time distribution is uniform (random variable *X*_2_). When not stated differently, the parameters used are $1/\alpha =5,\;\mu^{*}=5$. Fig 3 shows the results of comparison of $\rho^{*}$ influence on *Z*_*n*_(*t*) and $Z_{n}^{*}(t)$ in different scenarios. One can notice that the mean number of finished jobs decreases with the increase of $\rho^{*}.$ In Fig 3d we can observe that the mean number of jobs finished before *t* increases visibly faster when $\rho^{*}\leq 1$ than for $\rho^{*}>1$.

**Fig 4:**
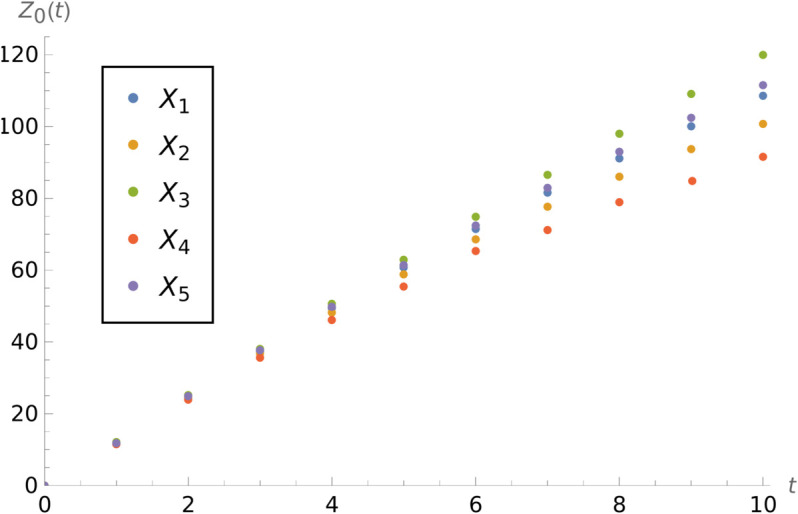
Influence of *X.* *Z*_0_(*t*) for different interarrival distributions.

**Example 4.** Let us consider a model with $1/\alpha =1,\;mu=40,\text{ and }\mu^{*}=15.$ In the Fig 4 we can see the plots of *Z*_0_(*t*) for different input streams. As the distribution of interarrival times, the distributions listed in the table were used. We can observe that, in time, the difference between mean number of finished jobs rises. The least expected number of jobs finished prior to *t* is for the interarrival distribution with the lowest coefficient of variation. For the distribution with the largest coefficient of variation, i.e., the gamma distribution, the expected number of finished jobs is also the largest.

## Conclusions and future work

In this paper, a *GI*/*M*/1/*N* queue with single WV was analyzed. The Laplace-Stieltjes transform of the generating function of the time-dependent distribution of the number of jobs finished before a given moment *t* was found. With the use of the numerical Laplace transform inversion algorithm, the mean number of finished jobs was computed in different settings to compare the characteristic of interest for different model parameters and to evaluate the impact of those parameters on the queue behavior.The results showed that the productivity of the model depends strongly on the system settings. Another transient characteristics (e.g., distribution of lost jobs) of this kind of model are left for future research, as well as the analysis of a model with multiple WV or a model with input stream that exhibit some kind of dependency.

## Supporting information

S1 Plot data Data presented on plots in numerical study section. (CSV)
